# *Temnothorax crasecundus* sp. n. – a cryptic Eurocaucasian ant species (Hymenoptera, Formicidae) discovered by Nest Centroid Clustering

**DOI:** 10.3897/zookeys.479.8510

**Published:** 2015-01-29

**Authors:** Bernhard Seifert, Sandor Csösz

**Affiliations:** 1 Senckenberg Museum for Natural History Goerlitz, Am Museum 1, D - 02826 Goerlitz, Germany; 2 MTA-ELTE-MTM, Ecology Research Group, Pázmány Péter sétány 1C, H-1117

**Keywords:** Numeric morphology-based alpha-taxonomy, Pragmatic Species Concept, parapatric species, hybridization, intraspecific dimorphism

## Abstract

The paper integrates two independent studies of numeric morphology-based alpha-taxonomy of the cryptic ant species *Temnothorax crassispinus* (Karavajev, 1926) and *Temnothorax crasecundus* **sp. n.** conducted by different investigators, using different equipment, considering different character combinations and evaluating different samples. Samples investigated included 603 individual workers from 203 nests – thereof 104 nest samples measured by Seifert and 99 by Csösz. The material originated from Europe, Asia Minor and Caucasia. There was a very strong interspecific overlap in any of the 29 shape characters recorded and subjective expert determination failed in many cases. Primary classification hypotheses were formed by the exploratory data analysis Nest Centroid (NC) clustering and corrected to final species hypotheses by an iterative linear discriminant analysis algorithm. The evaluation of Seifert’s and Csösz’s data sets arrived at fully congruent conclusions. NC-Ward and NC-K-means clustering disagreed from the final species hypothesis in only 1.9 and 1.9% of the samples in Seifert’s data set and by 1.1 and 2.1% in Csösz’s data set which is a strong argument for heterospecificity. The type series of *Temnothorax crassispinus* and *Temnothorax crasecundus* **sp. n.** were allocated to different clusters with p = 0.9851 and p = 0.9912 respectively. The type series of the junior synonym *Temnothorax slavonicus* (Seifert, 1995) was allocated to the *Temnothorax crassispinus* cluster with p = 0.9927. *Temnothorax crasecundus* **sp. n.** and *Temnothorax crassispinus* are parapatric species with a long contact zone stretching from the Peloponnisos peninsula across Bulgaria northeast to the southern Ukraine. There is no indication for occurrence of interspecifically mixed nests or intraspecific polymorphism. However, a significant reduction of interspecific morphological distance at sites with syntopic occurrence of both species indicates local hybridization. The results are discussed within the context of the Pragmatic Species Concept of Seifert (2014). The taxonomic description and a differential diagnosis of *Temnothorax crasecundus* **sp. n.** are given.

## Introduction

The small Formicoxenine ants *Temnothorax nylanderi* (Förster, 1850) and *Temnothorax crassispinus* (Karavajev, 1926) are dominant elements of the forest floor fauna of European temperate woodland biomes. They have repeatedly stood in the focus of thorough studies after Seifert (1995) presented evidence of their distinctness. In that paper he treated the two entities as subspecies: *Leptothorax nylanderi nylanderi* (Förster, 1850) and *Leptothorax nylanderi slavonicus* Seifert, 1995. He concluded that they spread from an Ibero-Italian and Balkan Pleistocene refuge respectively and have met in Germany along a 900-kilometers-long front line latest during the Atlantic (about 7500 years before present). In the years 1990–2000, this front-line ran in northeast Germany over Anklam-Krakow am See-Osterburg-Burg-Aken-Leipzig-Grimma-Freiberg and in south Germany over Pegnitz-Ingolstadt-München. After objections against the use of the subspecies concept (pers. comm. of Barry Bolton to Seifert in 1995), both entities were treated and named as parapatric species (Seifert 1996). Based on geographic and morphological indications, Radchenko (2000) later supposed a junior synonymy of *Leptothorax slavonicus* with *Leptothorax crassispinus* Karavajev, 1926. After getting access to the type series of *Leptothorax crassispinus* kept in the museum of Kiev, Seifert (2007) confirmed Radchenko’s view and transferred the species to the genus *Temnothorax* Mayr, 1861 in agreement with the new genus-level classification of Bolton (2003).

According to mtDNA data (Pusch et al. 2006), the splitting of *Temnothorax nylanderi* and *Temnothorax crassispinus* in separate glacial refuges should have occurred already in Early Pleistocene ± 1.4 Myr b.p. if 2.0% sequence divergence correspond to 1 Myr. These refuge areas probably have been used with similar topography and migration routes during all glaciations since then. A very low within-species mtDNA sequence divergence (0.14% in *Temnothorax nylanderi* and 0.29% in *Temnothorax crassispinus*) indicates a rapid postglacial spreading.

Frequent hybridization of *Temnothorax nylanderi* and *Temnothorax crassipinus* along the front-line in Germany has been shown (Pusch et al. 2006) or made credible (Seifert 1995). The depth of the front zone is not larger than 25 km while the east-west extension of both species’ range is 3600 km at least. Despite some 7500 years of contact, the genomic integrity of the species and the clear-cut parapatric separation has been maintained by apparently two factors: (a) there is a significant selection against hybrid genotypes – hybrid gynes are smaller and show a strongly reduced fertility (Pusch et al. 2006) and (b) mating flights are short-ranged and there is no long-range postmating dispersal of gynes (Plateaux 1986, Foitzik and Heinze 2001).

Very early it became obvious to the senior author (Seifert) that there existed a third cryptic entity of the *Temnothorax nylanderi* complex in the Balkans, Asia Minor and Caucasus that may occur syntopic with *Temnothorax crassispinus*. These preliminary investigations, however, came to a complete standstill after three rejections of research funding proposals in the years 2007–2009. More recently, the junior author (Csösz) could investigate further material from the Balkans and Asia Minor. Csösz’s data considerably improved the knowledge about the relations of the two species in the contact zone. We fuse in this paper the independent approaches of two investigators to a broad basis of evidence for the existence of the new cryptic species *Temnothorax crasecundus* sp. n. which is a sibling species of *Temnothorax crassispinus*. The contact situation is comparable to that found in *Temnothorax nylanderi* and *Temnothorax crassispinus*. *Temnothorax crasecundus* sp. n. and *Temnothorax crassispinus* are parapatric – the front line in Greece and Bulgaria is known and predicted to run diagonally through Romania and Moldova.

*Temnothorax crasecundus* sp. n. and *Temnothorax crassispinus* are truly cryptic species. Each of the 29 investigated shape characters and absolute size show a considerable interspecific overlap even in nest sample means and much more so on individual level. Furthermore, there is no clear signal provided by pigmentation and overall phenotypic impression. Both authors, having a long experience in identification of *Temnothorax* ants by simple eye inspection, are able to make a fair subjective guess on the species identity in a good number of samples but fail in many others. Both species thus fully fit the definition of cryptic species by Seifert (2009): “...*species which are not safely separable by primary visual or acoustic perception of an expert. This reflects the immediate sense of the word and restricts the term to the truly cryptic cases – i.e., to species not safely separable by training of innate pathways of the human cognitive system.*”

Morphology is essential to establish the link between species delimitation and Linnean nomenclature (Franz 2005, Brower 2006, Schlick-Steiner et al. 2007, Steiner et al. 2009). Allocation of taxonomic names to cryptic species is only possible via direct investigation of type specimens – in our case, 90-years old types of some 2.7 millimeters-long ants and other still older museum specimens have to be evaluated. Degradation of DNA is very likely and the success even of advanced methods of DNA analysis unpredictable. A curator of a museum collection should not allow destructive DNA sampling from a type specimen of a small insect and there is no doubt that next generation sequencing in our 600 dry mounts would be enormously costly and time-consuming. Much more practicable are non-destructive, advanced methods of morphological clustering. The methodology of numeric morphology-based alpha-taxonomy (NUMOBAT, Seifert 2009) in ants experienced a fast evolution during the last years. It started with hypothesis-driven analyses of conventional linear morphometrics (Seifert 2005). Later, explorative analyses of more complex data sets (Seifert et al. 2009, Seifert 2011) and geometric morphometrics (Bagherian et al. 2012, Seifert et al. 2014) were added to the toolbox. The most recent methodological improvement is Nest-Centroid clustering (NC clustering, Seifert et al. 2013). NC clustering is a high-resolution explorative data analysis applicable to any group of eusocial organisms or to any cohesive biological system providing repeats of definitely conspecific elements.

In this paper, we firstly present our argumentation why *Temnothorax crasecundus* sp. n. has a separate species status and why local hybridization is no argument against heterospecificity. Then we give the formal description of the new species.

## Material and methods

### Material

A total of 203 nest samples with 603 individual workers of both species was investigated – 104 samples by Seifert and 99 by Csösz. Only one nest series, the type series of *Temnothorax crassispinus* (Karavajev, 1926), was investigated by both Csösz and Seifert. Though genetically representing the same nest population we treated these two samples as operationally different because of the deviating character systems of the investigators and their different individual selection of workers from the type series.

### Temnothorax crassispinus

A total of 119 nest samples originated from the following countries: Austria 13, Bosnia 2, Bulgaria 7, Czechia 3, Germany 39, Greece 24, Hungary 8, Italy 2, Macedonia 3, Moldova 2, Poland 5, Serbia 1, Slovenia 4 and Ukraine 7 samples. A detailed account of the samples is given in the following under the sequence site, date in the format yyyy.mm.dd, sample No [latitude in decimal format, longitude in decimal format, altitude].

**AUSTRIA:** Arnoldstein, 1994.06.03, [46.550 N, 13.704 E, 570 m]; Bad Vöslau, pre 1980 [47.967 N, 16.222 E, 250 m]; Einöd-0.5 km N, 1994.08.05, No 40 [48.318 N, 15.732 E, 250 m]; Göttlesbrunn, 1955.08.25, [48.058 N, 16.738 E, 170 m]; Innsbruck, Kalvarienberg, 1994.08.05, No H2 [47.283 N, 11.435 E, 630 m]; Innsbruck, 1944.04.02 [ 47.266 N, 11.408 E, 580 m]; Kärnten: Gösselsdorfer See, 1994.08 [46.566 N, 14.618 E, 680 m]; Paudorf-1 km N, 1994.07.13, No 41, g82 [48.358 N, 15.618 E, 350 m]; Roppen-1 km E, 1994.07.04, No 207 [47.223 N, 10.831 E, 730 m]; Seebenstein: Türkensturz, pre 1970 [47.679 N, 16.136 E, 540 m]; Wachau: Spitz-5 km NW, 1990.07.01, No 2774 [48.392 N, 15.358 E, 400 m]; Wellersdorf-2 km NW, 1994.07.11 [46.567 N, 14.169 E, 770 m]. **BOSNIA:** Golubic-10 km N, 1989.09.15, No 51, 56 [44.768 N, 15.929 E, 400 m]. **BULGARIA:** Kokalyane-3 km E, 2009.06.06, No 080, 1033, 1035 [42.579 N, 23.434 E, 650 m]; Vitosha: Vladaya district, 2004, No 590, 856 [42.630 N, 23.205 E, 930 m]; Vitosha: Zheleznitsa- 1 km N, 2009.06.06, No 1009 [42.546 N, 23.365 E, 1000 m]; Vratehansica Planina: Vratsa-5 km S, 2009.06.07, No 1074 [43.137 N, 23.591 E, 1000 m]. **CZECHIA:** Masovice-3.7 km S, 1997.09.18, No 227 [48.824 N, 15.977 E, 285 m]; Strelna-0.5 km E, 1993.09.28, No 02, 03 [50.667 N, 13.755 E, 350 m]. **GERMANY:** Abensberg-10 km E, 2000.09.05, No AB1, AB2 [48.812 N, 11.969 E, 425 m]; Adlersberg- 0.5 km W, 2000.09.06, No AD/M6, AD/M7 [49.042 N, 12.004 E, 453 m]; Altenhain-1 km NW, 1994.06, No 07, 08 [51.299 N, 12.685 E, 15 m]; Berching, 2001.09, No 1, 3 [49.115 N, 11.444 E, 410 m]; Berzdorf, 1981.04.07 [51.055 N, 14.886 E, 274 m]; Berzdorf, 1993.03.19, No g31 [51.055 N, 14.886 E, 274 m]; Genthin-3.6 km S, 1994.06, No 6, 8, 04 [52.375 N, 12.150 E, 39 m]; Glesien-1.6 km W, 1994.06, No 3, 5 [51.445 N, 12.205 E, 122 m]; Hohburg-1 km S, 1993.06.19, No g77 [51.405 N, 12.799 E, 170 m]; Koldenhof, 2000.11.02, No-000074 [53.330 N, 13.340 E, 119 m]; Kühren-1 km W, 1993, No 94 [51.875 N, 11.978 E, 58 m]; Löbauer Berg, 1983.07.13 [51.089 N, 14.692 E, 392 m]; Meissen, Bosel, 1982.06.09, No 303 [51.138 N, 13.514 E, 170 m]; Mertitz, 2001.06.25 [51.177 N, 13.321 E, 138 m]; Obergruna-1 km S, 1993.04.12, No 59, 145 [51.005 N, 13.316 E, 285 m]; Ponholz-2 km SE, 2000.09.06, No P10, P11 [49.151 N, 12.119 E, 399 m]; Quolsdorf-2.8 km NE, 1992.04.06 [51.402 N, 14.872 E, 115 m]; Serrahn, 2000.05.04, No 000036, 17 [53.670 N, 12.350 E, 62 m]; Steffenhagener Heide, 2007.04.27 [54.110 N, 13.295 E, 7 m]; Technitz-1.2 km WNW, 1994.06, No 1, 6, 8 [51.130 N, 13.051 W, 165 m]; Trebsen-2.1 km SW, 1994, No 02, 03, 08, 09, 44 [51.272 N, 12.697 E, 165 m]; Uchtspringe-0.5 km S, 1995, No 10 [52.534 N, 11.605 E, 75 m]. **GREECE:** Chelmos, 1994.06.04, No 1394 [37.987 N, 22.198 E, 2000 m]; Kalamata-20 km E, 1994.06.01, No 1348, 1349 [37.080 N, 22.280 E, 1250 m]; Kastanitsa-4 km SW; 2000.04.22, No 20 [37.280 N, 22.670 E, 1300 m]; Konitsa-5 km N, 1996.05.23, No 355 [40.108 N, 20.764 E, 550 m]; Levidi-10 km S, 2000.04.27, No 118, 119, 137 [37.630 N, 22.280 E, 1700 m]; Meliana vic., 1996.05.21, No 024 [39.361 N, 20.787 E, 700 m]; Parnon Mts., 2011.04.26, No 117, 145 [37.270 N, 22.610 E, 1650 m]; Sitena vic., 2000.04.05, No 56 [37.300 N, 22.650 E, 1000 m]; Sitena-3 km W, 2000.04.25-66, 071,079 [37.300 N, 22.600 E, 1700 m]; Sparti-20 km SW, 2000.04.29, No 150b, 150c [36.970 N, 22.350 E, 1950 m]; Taigetos Oros, 2011.04.30, No 305 [36.948 N, 22.377 E, 1500 m]; Tripolis-15 km NNW, 2011.04.29, No 214, 221 [37.629 N, 22.302 E, 1000 m]; Tripolis-22 km NNW, 2011.04.29, No 234, 238 [37.654 N, 22.268 E, 1600 m]; Vamvakou-3 km SE, 2011.04.26, No 162 [37.22 N, 22.59 E, 1300 m]; Vitina-5 km NE, 2011.04.29, No 261 [37.681 N, 22.208 E, 1000 m]. **HUNGARY:** Budapest, 1909.05.21 [47.543 N, 18.966 E, 250 m]; Csillebérc, 2004.05.30, No 397 [47.500 N, 18.959 E, 400 m]; Hatvan, 2011.03.12, No 432, 433 [47.673 N, 19.647 E, 200 m]; Isaszeg, 2011.04.02, No 437 [47.535 N, 19.397 E, 200 m]; Pécs (leg. Kaufmann), pre 1945 [46.104 N, 18.245 E, 200 m]; Vérteskozma, 2009.04.30, No 411 [46.459 N, 18.432 E, 350 m]. **ITALY:** Villa Santina, 1989.09.18, No 03, 04 [46.403 N, 12.844 E, 460 m]. **MACEDONIA:** Jacupitsa, 2009.06.19, No 001 [41.418 N, 21.416 E, 1300 m]; Konopishte, 2009.06.19, No 003 [41.248 N, 22.079 E, 672 m]; Lukovo, 2009.06.16, No 004 [41.366 N, 20.606 E, 590 m]. **MOLDOVA:** Vall du Berlad (Barlad Valley), pre 1930, No 1, 2 [47 N, 28 E, 70 m]. **POLAND:** Kielce: Pongrac [50.83 N, 20.66 E, 300 m]; Osiecznica-3 km W, 1994.04.09, No 85, 104 [51.344 N, 15.379 E, 187 m]; Wolin: Wapnica-3.5 km E, 2006, No 46, 79 [53.883 N, 14.485 E, 35]. **SERBIA:** Sremska Kamenica, 1971.05.02 [45.22 N, 18.44 E, 300 m]. **SLOVENIA:** Knezac, 1989.09.17, No 10, 11 [45.619 N, 14.250 E, 615 m]; Postoijna-12 km W, 1997.05.29, No 401 [45.778 N, 14.062 E, 900 m]; Novo Mesto, 2007.05.29, No WL16 [45.811 N, 15.169 E, 200 m]. **UKRAINE:** Cherkassy: Kanev Nat. Res. [49.711 N, 31.477 E, 220 m]; Donetzk, S riv. Donetz, 1982.07.16 [47.9 N, 37.8, 120 m]; Golosseyev, pre 1926 [50.5 N, 30.5 E, 140 m]; Kiev vic., 1988.07.08 [50.5 N, 30.5 E, 140 m]; Pervomaijsk, 1998.06.10 [48.044 N, 30.859 E, 100 m]; Pischa, 2004.06.10 [51.071 N, 21.003 E, 170 m].

### *Temnothorax crasecundus* sp. n.

A total of 84 nest samples originated from the following countries: Armenia 1, Bulgaria 31, Georgia 6, Greece 15, Romania 4, Russia 2, Turkey 21 and Ukraine 4 samples. A detailed account of the samples is given in the following.

**ARMENIA:** Armenia: without site, 1986.06.11 [40 N, 45 E, 1600 m]. **BULGARIA:** Arkutino, 1978.08.01 [42.33 N, 27.77 N, 15 m]; Bistrits-1 km N, 2009.06.06, No 1004 [42.594 N, 23.363 E, 400 m]; Dobrostan, 1982.09.12-55 [41.93 N, 24.88 E, 1500 m]; Dospat-2SW, 2009.0610, No 1203 [41.634 N, 24.149 E, 1300 m]; German Monastery, 2004.05, No 467, 470 [42.602 N, 23.434 E, 800 m]; Harsovo-1 km SE, 2009.06.09, No 1130, 1130/1, 1131 [41.458 N, 23.390 E, 200 m]; Kiten, 2011.04.29, No 373 [42.238 N, 27.772 E, 27 m]; Kokalyane-3 km E, 2009.0606, No 079 [42.579 N, 23.434 E, 650 m]; Malko Tarnovo: Propada, 2009.07.26, No 370 [41.982 N, 27.492 E, 385 m]; Malko Tarnovo: Brashlyan 2009.08.22, No 374 [42.044 N, 27.427 E, 340 m]; Mladezhko, 2009.08.21, No 366 [42.152 N, 27.362 E, 220 m]; Novakovo-2 km SE, 2009.06.12, No 1299 [41.887 N, 25.099 E, 400 m]; Obsor, 1981.08.01 [42.82 N, 27.86 E, 50 m]; Pasarel-1 km NW, 2009.06.06, No 070, 078 [42.594 N, 23.362 E, 770 m]; Peshtera, 2008.05.25, No 369 [42.297 N, 24.299 E, 580 m]; Pirin Mts.: Rozen-8 km N, 1982.09.05, No 0, 396 [41.60 N, 23.45 E, 1400 m]; Plovdiv, 1977.05.27 [42.14 N, 24.72 E, 200 m]; Sofia: Borisova Park 1, 2004, No 841 [42.680 N, 23.342 E, 596]; Sofia: Borisova Park 2, 2004, No 509, 558 [42.678 N, 23.351 E, 586]; Sofia: Lozenetz distr., 2004, No 709 [42.666 N, 23.312 E, 620 m]; Tsaparevo-2 km S, 2009.0609, No 1168 [41.612 N, 23.097 E, 800 m]; Vitosha: Vladaya distr., 2004, No 596, 854 [42.630 N, 23.205 E, 930 m]; Zeleznitza-1 km N, 2009.06.06, No 043, 047 [42.539 N, 23.367 E, 1000 m]. **GEORGIA:** Abastumani-1.4 km W, 2013.09.17, No GEOII-70, GEOII-71, GEOII-72 [41.758 N, 42.817 E, 1532 m]; Daba-0.26 km E, 2010.08 [41.811 N, 43.452 E, 1030 m]; Pizunda, 1984.08.11 [43.153 N, 40.342 E, 17 m]; Sedaseni-Kloster, 2004.07.28 [41.871 N, 44.768 E, 1150 m]. **GREECE:** Karitza-6 km W, 1998.04.04 [39.840 N, 22.710 E, 750 m]; Kastanitza-6 km W, 1998.04.04 [37.28 N, 22.67 E, 1300 m]; Kosmas-2 km SW, 2000.04.26, No 93, 96, 098 [37.080 N, 22.730 E, 1100 m]; Levidi-10 km S, 2000.04.27, No 132 [37.630 N, 22.280 E, 1700 m]; Litohoro-3 km W, 1996.05.13 [40.080 N, 22.450 E, 1200 m]; Litohoro-7 km W, 1996.05.13, No 187 [40.112 N, 22.480 E, 600 m]; Parnon Mts., 2011.04.26, No 152 [37.27 N, 22.61 E, 1650 m]; Sitena-3 km W, 2000.04.25, No 62, 71a [37.300 N, 22.600 E, 1700 m]; Sparti-20 km SW, 2000.04.25, No 149 [36.97 N, 22.35 E, 1950 m]; Stagira, 2011.04.09, No 027 [40.531 N, 23.720 E, 585 m]; Taigetos Oros, 2011.04.30, No 302, 311 [36.948 N, 22.377 E, 1500 m]. **ROMANIA:** Comana: Vlasca, pre 1935, No 1, 2 [43.90 N, 28.31 E, 130 m]; Dobrogea: Babadag, 2005.06.02, No 105, 106 [44.857 N, 28.691 E, 100 m]. **RUSSIA:** Gelendzhik-5 km SSE, 2006.06.04, No 249 [44.48 N, 38.145 E, 150 m]; Obilnoje, 2006.06.08, No 219 [44.207 N, 43.538 E, 240 m]. **TURKEY:** Aydogdu-5 km SW, No 1147, 1157, 1158 [40.714 N, 42.495 E, 1500 m]; Cat-2 km S, 2012.07.02, No 046 [39.418 N, 35.957 E, 1550 m]; Demirköy, 2009.07.06, No 474, 476 [41.818 N, 27.814 E, 170 m]; Erzincan-25 km SE, 2012.07.13, No 179 [39.661 N, 39.734 E, 1200 m]; Eskishir-Sögüt, 2003.05.10, No 162 [39.550 N, 30.130 E, 966 m]; Ispir-10 km NW, 1993.07.01, No 1197 [40.585 N, 40.852 E, 1700 m]; Mahya hill, 2005.05.30, No 472 [41.771 N, 27.638 E, 820 m]; Mezraa vic., 2012.07.11, No 153, 159 [39.378 N, 39.805 E, 1300 m]; Ordu-25 km NW, 2012.07.21, No 396 [41.064 N, 37.711 E, 400 m]; Ordu-40 km WSW, 2012.07.21, No 402 [40.719 N, 37.622 E, 1000 m]; Posof-3 km E, 2012.07.17, No 303 [41.414 N, 42.762 E, 1500 m]; Pülümür vic., 2012.07.11, No 159 [39.482 N, 39.890 E, 1600 m]; Seydiler-7 km N, 2012.07.23, No 495 [41.694 N, 33.718 E, 1200 m]; Sögüt vic., 2003.05.10, No 166 [39.570 N, 30.13 E, 1050 m]; Tortum-15 km E: Kirecli Gecidi, 2012.07.13, No 205 [40.353 N, 41.704 E, 2400 m]; Tortum-45 km NNE, 2012.07.11, No 217 [40.325 N, 41.572 E, 1600 m]; Ulu Dag: Sogukpinar, 1993.07.05, No 1230 [40.055 N, 29.120 E, 750 m]. **UKRAINE:** Agarmis, 1980.09.29 [45.252 N, 35.025 E, 600 m]; Armiansk, 1985.05.04 [46.107 N, 33.693 E, 15 m]; Krasnolesye, 1980.09.12 [44.952 N, 34.102 E, 100 m]; Simferopol, 1995.08.13, No 826 [44.938 N, 34.099 E, 300 m].

### Type material

**Leptothorax nylanderi var. crassispina Karavajev, 1926**

The type series, stored in Shmalhausen Institute of Zoology Kiev and certainly representing a nest sample, is labeled “Kiev: Golosejev (Karavaijev No 3057)”. Three syntype workers were investigated by Seifert and seven syntype workers by Csösz.

***Leptothorax nylanderi slavonicus* Seifert, 1995**

The paratypes, seven workers on the same pin and originating from the nest that contained the queen holotype, are labeled “Kr. Görlitz, 19.3.1993, Schönau-Berzdorf, Hutberg, g31”, „Leptothorax nylanderi slavonicus Seifert”, “Paratypes”. Four paratypes of this sample were investigated by Seifert. Material is stored in Senckenberg Museum of Natural History Görlitz. Csösz investigated four worker paratypes of another nest series from the type locality, labelled “Germany, Kr. Görlitz, Hutberg Schönau-Berzdorf, 19.03.1993 Seifert”.

***Temnothorax crasecundus* sp. n.**

The worker holotype is labelled “BUL: 42.6785°N, 23.3508°E Sofia, 586 m, Borisova gradina Park, Part 2 V.Antonova 2004.05-509” and “Holotype *Temnothorax crasecundus* Seifert & Csösz”. Four worker paratypes, two males and two gynes from the holotype nest are mounted on two other pins and carry the same collecting data label and “Paratypes *Temnothorax crasecundus* Seifert & Csösz”. A second series with five worker paratypes on two pins is labeled “BUL: 42.6797°N, 23.3417°E Sofia, 596 m, Borisova gradina Park, Part 1 V.Antonova 2004.05-841” and “Paratypes *Temnothorax crasecundus* Seifert & Csösz”. These two type series are stored in Senckenberg Museum of Natural History Görlitz. A third paratype series containing three workers on the same pin, is labelled “Bulgaria_28: East Rhodopes, 2 km SE Novakovo 25 km SE. Asenovgrad, 1299, 400mH, 41°53'12"N, 25°5'55"E, 12.06.2009, Leg A Schulz”, “ANTWEB CASENT 0906045“ and is stored in the Hungarian Museum of Natural History Budapest.

### Methods

The senior and junior author performed two independent investigations of worker ant morphology, considering different character combinations and using different microscopic equipment. Seifert recorded 18 and Csösz 22 primary morphometric characters. In bilaterally developed characters, arithmetic means of both body sides were calculated. All measurements were made on mounted and fully dried specimens. Measurements of body parts always refer to real cuticular surface and not to the diffuse pubescence surface.

### Equipment and measurement procedures of Seifert

Seifert used for spatial adjustment of specimens a pin-holding stage, permitting full rotations around X, Y, and Z axes and a Leica M165C high-performance stereomicroscope equipped with a 2.0 planapochromatic objective (resolution 1050 lines/mm) at magnifications of 120–384×. The mean relative measuring error over all magnifications was 0.3%. A Schott KL 1500 cold-light source equipped with two flexible, focally mounted light-cables, providing 30°-inclined light from variable directions, allowed sufficient illumination over the full magnification range and a clear visualization of silhouette lines. A Schott KL 2500 LCD cold-light source in combination with a Leica coaxial polarized-light illuminator provided optimal resolution of tiny structures and microsculpture at highest magnifications. Simultaneous or alternative use of the cold-light sources depending upon the required illumination regime was quickly provided by regulating voltage up and down. A Leica cross-scaled ocular micrometer with 120 graduation marks ranging over 52% of the visual field was used. To avoid the parallax error, its measuring line was constantly kept vertical within the visual field.

Equipment and measurement procedures of Csösz

Measurements were made using a pin-holding stage, permitting rotations around X, Y, and Z axes. An Olympus SZX9 stereomicroscope was used at a magnification of 150× for each character, allowing a precision of ± 2 µm.

### The morphometric characters

29 morphometric characters were investigated – ten of these by both authors. We give the character definitions in alphabetic order. In square brackets is indicated who investigated a character.

CL [Csösz & Seifert]: maximum cephalic length in median line; the head must be carefully tilted to the position with the true maximum. Excavations of hind vertex and/or clypeus reduce CL.

CS [Seifert]: cephalic size; the arithmetic mean of CL and CW, used as a less variable indicator of body size.

CSb [Csösz]: cephalic size; the arithmetic mean of CL and CWb.

CW [Seifert]: maximum cephalic width; the maximum is found in *Temnothorax* and *Leptothorax* usually across and including the eyes, exceptionally posterior of the eyes.

CWb [Csösz]: maximum width of head capsule, measured just posterior of the eyes.

EL [Csösz]: maximum diameter of the eye.

EYE [Seifert]: eye-size index: the arithmetic mean of the large (EL) and small diameter (EW) of the elliptic compound eye is divided by CS, i.e. EYE=(EL+EW)/(CL+CW). All structurally visible ommatidia are considered.

**Figures 1–8.**
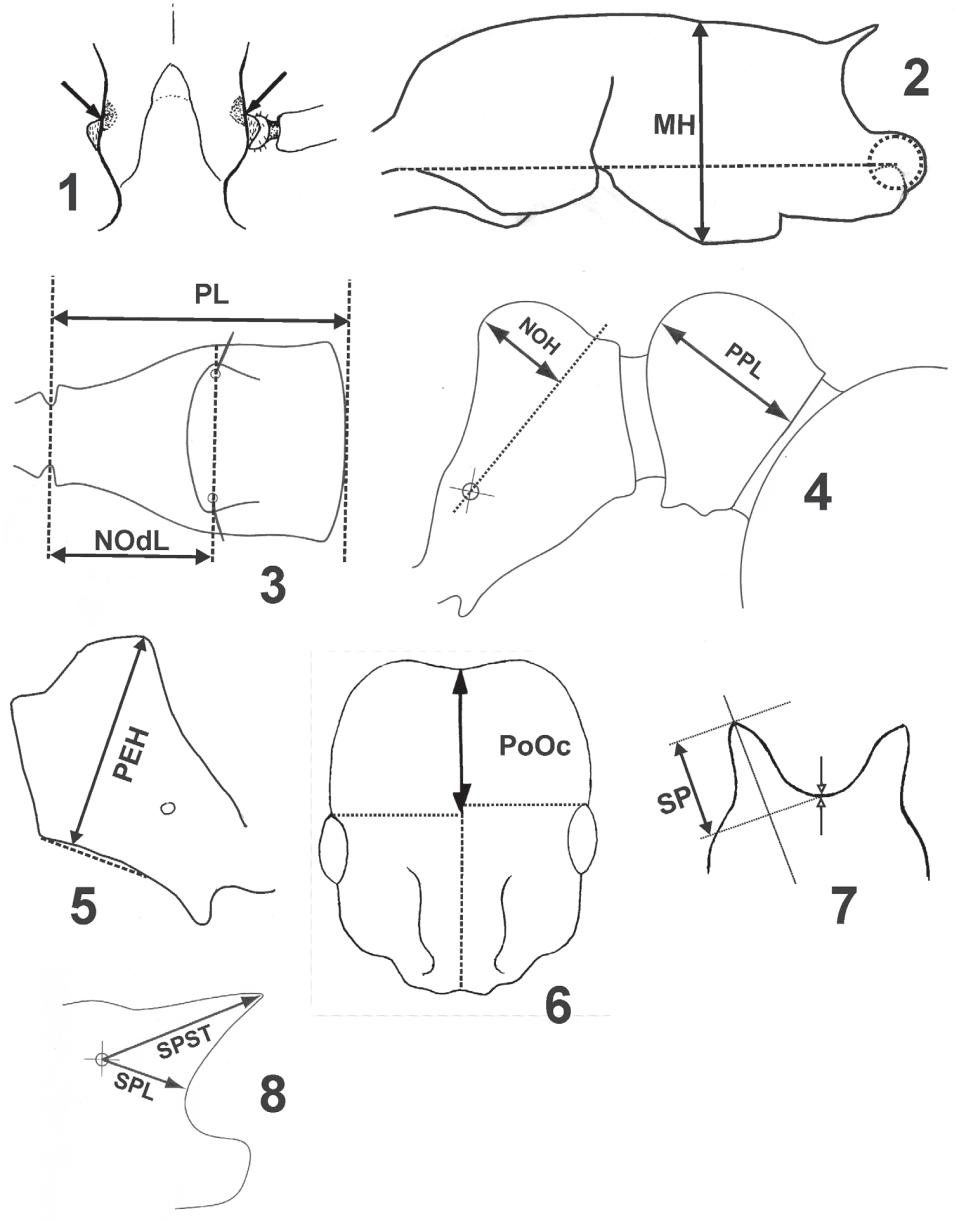
Explanation of morphometric characters.

FRS [Csösz & Seifert]: distance of the frontal carinae immediately caudal of the posterior intersection points between frontal carinae and the lamellae dorsal of the torulus (arrows in Fig. 1). If these dorsal lamellae do not laterally surpass the frontal carinae, the deepest point of scape corner pits may be taken as reference line. These pits take up the inner corner of scape base when the scape is fully switched caudad and produce a dark triangular shadow (spotted area in Fig. 1) in the lateral frontal lobes immediately posterior of the dorsal lamellae of scape joint capsule.

MGr [Seifert]: depth of metanotal groove or depression, measured from the tangent connecting the dorsalmost points of promesonotum and propodeum; here given as per cent ratio of CS.

MH [Seifert]: in workers: with mesosoma in lateral view and measured orthogonal to “longitudinal mesosomal axis”, MH is the longest measurable ***section*** line of mesosoma at mesopleural level (not height above all). “Longitudinal mesosomal axis” in lateral view is defined as straight line from the centre of propodeal lobe (centre of circus in Fig. 2) to the border point between anterior pronotal shield and propleuron. In gynes it is the longest section line directed perpendicular to the straight dorsal profile line of mesosoma (formed by mesonotum and scutellum). The lower measuring point is usually the lowest part of mesopleuron.

ML [Csösz & Seifert]: in workers: mesosoma length from caudalmost point of propodeal lobe to transition point between anterior pronotal slope and anterior propodeal shield (preferentially measured in lateral view; if the transition point is not well defined, use dorsal view and take the centre of the dark-shaded borderline between pronotal slope and pronotal shield as anterior reference point). In gynes: length from caudalmost point of propodeal lobe to the most distant point of steep anterior pronotal face.

MW [Csösz & Seifert]: maximum mesosoma width; this is in workers pronotal width, in gynes it is measured anteriorly of the tegulae.

NOdL [Csösz]: Anterior length of petiole measured in dorsal view. Distance from the (centre of anteriormost seta pit on the petiolar node to the level of the constriction of articulation condyle with propodeum (Fig. 3). Measuring requires a change of focus from above (seta pit) to below (constriction). Dorsal view is achieved when the dorsalmost point of anterior petiolar peduncle at the level of its strongest constriction and the dorsalmost point of caudal petiolar margin are in the same focal level.

NOH [Csösz]: Maximum height of the petiolar node, measured in lateral view from the uppermost point of the petiolar node perpendicular to a reference line set from the petiolar spiracle to the imaginary midpoint of the transition between the caudal slope and the caudal cylinder of the petiole (Fig. 4).

NOL [Csösz]: Length of the petiolar node, measured in lateral view from petiolar spiracle to dorso-caudal corner of caudal cylinder. Do not erroneously take as reference point the dorso-caudal corner of the helcium, which is sometimes visible.

PEH [Csösz & Seifert]: maximum petiole height. The chord (dashed line in Fig. 5) of ventral petiolar profile at node level is the reference line perpendicular to which the maximum height of petiole is measured.

PEL [Seifert]: diagonal petiolar length in lateral view; measured from anterior corner of subpetiolar process to dorsocaudal corner of caudal cylinder.

PEW [Csösz & Seifert]: maximum width of petiole.

PL [Csösz]: Total petiole length measured in dorsal view; distance between the dorsalmost point of caudal petiolar margin and the dorsalmost point of anterior petiolar peduncle at the transversal level of its strongest constriction. Positioning of petiole as in NOdL (Fig. 3).

PoOc [Csösz & Seifert]: postocular distance. Use a cross-scaled ocular micrometer and adjust the head to the measuring position of CL. Caudal measuring point: median occipital margin; frontal measuring point: median head at the level of the posterior eye margin. Note that many heads are asymmetric and average the left and right postocular distance (Fig. 6).

PPH [Csösz]: Maximum height of the postpetiole in lateral view measured perpendicularly to a line defined by the linear section of the segment border between dorsal and ventral petiolar sclerite.

PPL [Csösz]: Maximum length of the postpetiole measured in lateral view perpendicular to the straight section of lateral postpetiolar margin (Fig. 4).

PPW [Csösz & Seifert]: maximum width of postpetiole.

SL [Csösz & Seifert]: maximum straight line scape length excluding the articular condyle as arithmetic mean of both scapes.

SP [Seifert]: maximum length of propodeal spines; measured in dorsofrontal view along the long axis of the spine, from spine tip to a line, orthogonal to the long axis, that touches the bottom of the interspinal meniscus (Fig. 7). This mode of measuring is less ambiguous than other methods but it results in some spine length in species with reduced spines.

SPL [Csösz]: Minimum distance between the center of propodeal spiracle and the margin of subspinal excavation measured with both end points positioned in the same focal level (Fig. 8).

SPBA [Csösz & Seifert]: the smallest distance of the lateral margins of the spines at their base. This should be measured in dorsofrontal view, since the wider parts of the ventral propodeum do not interfere with the measurement in this position. If the lateral margins of spines diverge continuously from the tip to the base, a smallest distance at base is not defined. In this case, SPBA is measured at the level of the bottom of the interspinal meniscus.

SPST [Csösz & Seifert]: distance between the centre of propodeal stigma and spine tip. The stigma centre refers to the midpoint defined by the outer cuticular ring but not to the centre of real stigma opening that may be positioned eccentrically.

SPTI [Csösz & Seifert]: the distance of spine tips in dorsal view; if spine tips are rounded or truncated, the centres of spine tips are taken as reference points.

SPWI [Csösz]: Maximum distance between outer margins of spines; measured in same position as SPBA.

### Explorative and supervised data analyses and classification methods

The delimitation of the cryptic species was done by an interaction of Nest-Centroid Clustering (NC clustering) and a confirmative linear discriminant analysis (LDA). NC Clustering was run both as hierarchical NC-Ward clustering and non-hierarchical NC-K-means clustering. These methods were described in more detail by Seifert et al. (2013) who also provided a script written in R and freely available under the GNU / GPL license from the following website: http://sourceforge.net/projects/agnesclustering/.

The same mathematical procedures were applied for the data sets of Seifert and Csösz. NC-Ward clustering was run first to indicate the putative number of K main clusters. In the second step NC-K-Means was performed with the setting of K classes suggested by NC-Ward. Classifications being coincident between the hierarchical and non-hierarchical clustering formed the hypothesis for the controlling LDA that was subsequently run. Samples with classifications disagreeing between NC-Ward and NC-K-means were run in this LDA as wild-cards. The final classification (“final species hypothesis”) was established by the LDA in the iterative procedure described by Seifert et al. (2013). There remained no undecided cases independent which posterior probabilities they had. LDA and ANOVA tests were performed with the software package SPSS 15.0.

## Results and discussion

### Separation of the *Temnothorax nylanderi* species complex from other *Temnothorax* species

The W Palaearctic species of the *Temnothorax nylanderi* species complex – *Temnothorax nylanderi*, *Temnothorax crassispinus* and *Temnothorax crasecundus* sp. n. – can be separated from other species of the region by the following character combination.

1. Head short, mean index CL/CW only 1.053–1.063.
2. Whole dorsum of vertex regularly and continuously longitudinaly carinulate, shining surface areas are absent or restricted to a narrow median stripe.
3. Metanotal depression always visible, at least suggested.
4. Antennal club and femora never with a blackish pigmentation
5. Petiole in lateral view rather high and with a weakly concave frontal face; the anterior profiles of node and peduncle form an angle of about 150–155° whereas anterior and dorsal profiles of node form an angle of 90–105°. Dorsal profile of node steeply sloping down to caudal cylinder. The profiles of this slope and of the caudal cylinder form an angle of about 140°.
6. Propodeal spines acute, deviating from longitudinal axis of mesosoma by 32–42° and moderately long, SP/CS 0.200–0.260, SPST/CS 0.253–0.356.

### Convincing clustering of the cryptic species

**Figure 9.**
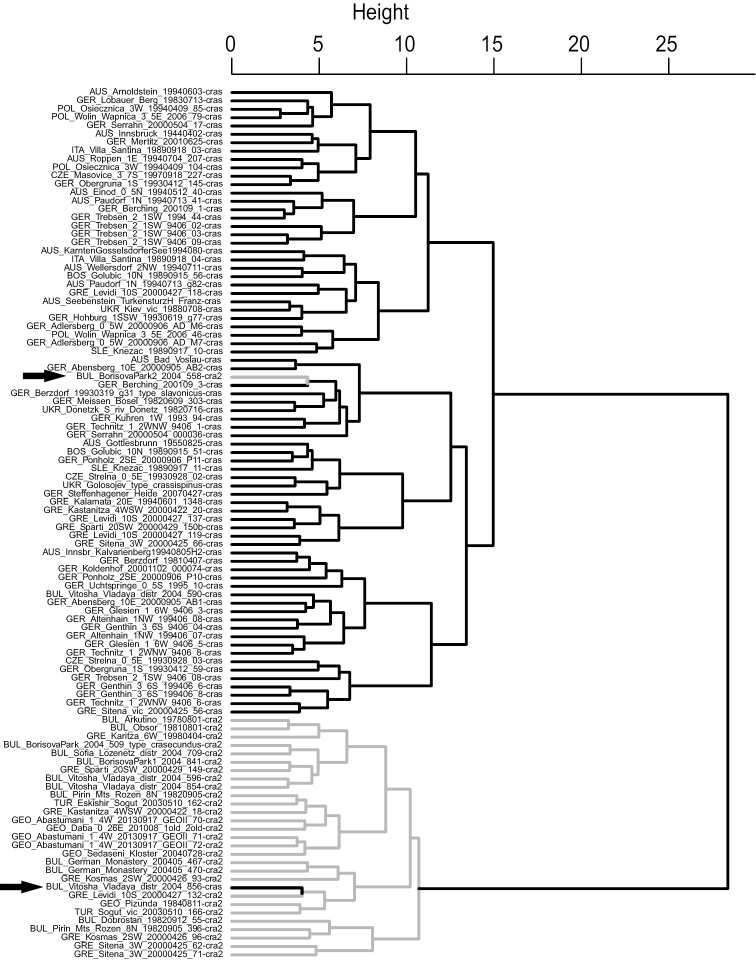
*Temnothorax crasecundus* sp. n. (grey branch) and *Temnothorax crassispinus* (black branch). NC-Ward clustering. Data set of Seifert: 104 nest samples investigated and 18 characters considered. Arrows point to samples clustered in disagreement with the final species hypothesis.

In the data set of Seifert and considering all 18 characters, NC Ward clustering provided a clear separation of the cryptic species in two main branches (Fig. 9). Accordingly, NC-K-means was run with K=2. The classifications of both NC-Ward and NC-K-means differed in 7.7% of samples. These samples were set as wild-cards in the controlling LDA. The final species hypothesis was determined in the iterative process described by Seifert et al. (2013). Both NC-Ward (Fig. 9) and NC-K-means clustering disagreed from the final species hypothesis in only 1.9% of samples. Character reduction by a stepwise LDA did not result in an improvement. The type series of *Temnothorax crassispinus* and *Temnothorax slavonicus* were allocated to the same cluster with p=0.9766 and p=0.9858 respectively while the holotype series of *Temnothorax crasecundus* sp. n. was allocated to the other cluster with p=0.9912.

**Figure 10.**
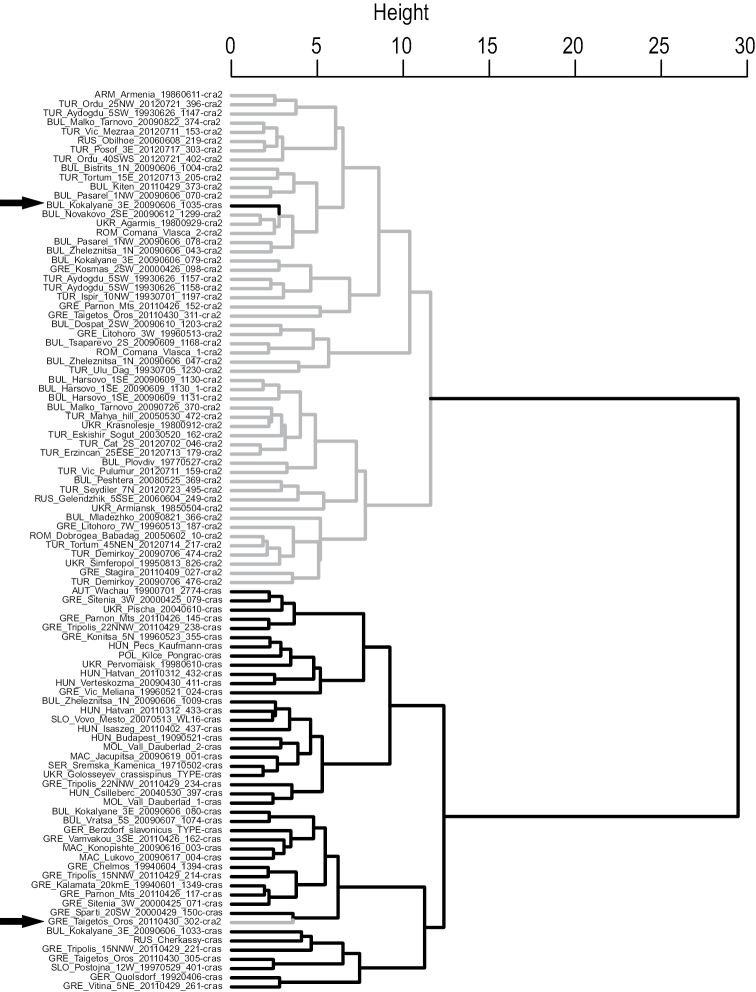
*Temnothorax crasecundus* sp. n. (grey branch) and *Temnothorax crassispinus* (black branch). NC-Ward clustering. Data set of Csösz: 99 nest samples and 10 characters considered. Arrows point to samples clustered in disagreement with the final species hypothesis. Figs 1 and 2 sum up to 203 different samples.

The results were similar in the data set of Csösz, considering all 22 characters and following the same procedure. The classifications of NC-Ward and NC-K-means differed in 11.1% of samples. NC-Ward disagreed from the final species hypothesis in 11.1% and NC-K-means in 3.1% of samples. A character reduction by a stepwise LDA, again performed iteratively, improved the classification result significantly. NC-Ward disagreed from the final species hypothesis in only 2.1% (Fig. 10) and NC-K-means in only 1.1% of samples when the ten characters CSb, CL/CWb, FRS/CSb, SL/CSb, ML/CSb, NOL/CSb, SPST/CSb, SPLV, PPW/CSb and SPWI/CSb are considered. The type series of *Temnothorax crassispinus* and the paratype series of *Temnothorax slavonicus* were allocated to the same cluster with p=0.9937 and p=0.9996 respectively while the third paratype series of *Temnothorax crasecundus* sp. n. from Bulgaria: Novakovo was allocated to the other cluster with p=0.9950.

**Figure 11.**
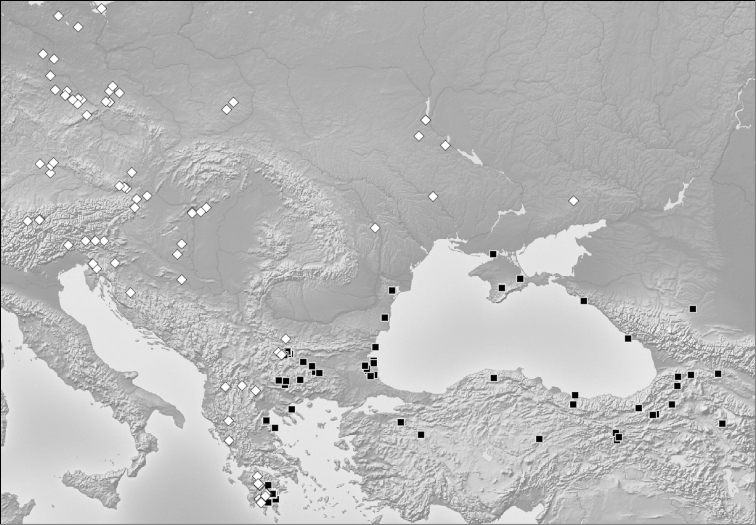
*Temnothorax crasecundus* sp. n. [black rectangles] and *Temnothorax crassispinus* [white rhombs]. The parapatric distribution is clearly shown.

We consider the congruent results of two independent investigations and investigators and the low disagreement of 1.1–2.1% between the classifications of exploratory data analyses with the final species hypothesis as a strong argument for heterospecificity of *Temnothorax crasecundus* sp. n. and *Temnothorax crassispinus*. This interpretation is supported by the coincidence of phenotyping with a clear-cut parapatric distribution (Fig. 11) and the rejection of intraspecific dimorphism (see below).

### Evolutionary divergence, local hybridization and heterospecificity

Seifert et al. (2013) reported an impressive performance of NC clustering in the separation of cryptic species but they also showed a significant weakness of this methodology if substantial numbers of hybrids are in a sample. In five data sets with a mean percentage of 18.9% hybrids, four of these did not involve cryptic species, the average agreement of NC-Ward and NC-K-means clustering with the LDA vector indication was reduced to 87.6%. They also stated that the best phenotypical identification of hybrids is given by their position along an interspecific vector (examples in Kulmuni et al. 2010; Seifert et al. 2010; Steiner et al. 2011; Bagherian et al. 2012). LDA vectors in particular are rather robust against the fluctuating nature of characters in hybrids. The larger the agreement of NC-clustering with the position along an interspecific LDA vector appears, the lower is the probability that hybrids are in the data set.

We achieved an agreement of 97.9–98.9% between the final species hypothesis and four different cluster analyses. This indicates a significant evolutionary divergence and could indicate that no or very few hybrids are present in our data set. Yet, the present problem is more difficult because extremely similar species are involved which prevents a reliable phenotypic identification of a particular hybrid sample. A broader statistic approach is needed. Accordingly, we compare the positions of samples along the interspecific LDA vector (i) from the potential contact zone, (ii) from the allopatric zones and (iii) from sites with established syntopic occurrence of both species. Reduced interspecific distances in contact zones are an indication that hybridization could have occurred. We consider the territories of Greece, Bulgaria, Romania and Moldova to form the potential contact zone (Fig. 11).

There is only a very weak, insignificant reduction of the distance from the zero point of the discriminant vector in samples from the potential contact zone compared to those from the allopatric zones (Tab. 1). However, there is a highly significant distance reduction in 31 samples from the nine Bulgarian and Greek sites with established syntopic occurrence of both species. This is in our opinion a strong suggestion of local hybridization and possibly also of introgression. We have determined all 203 samples following a YES/ NO decision but four samples, this is 2% of the whole material, are suspected to represent F1 hybrids or backcrosses of F1 hybrids with a parental species. However, as mentioned above, our phenotypical identification system does not allow a reliable identification of hybrids because few samples with discriminant values close to zero are also found in the allopatric ranges of both species where hybridization is impossible (Table 1).

**Table 1.** Distance of the sample means of *Temnothorax crassispinus* and *Temnothorax crasecundus* sp. n. from the zero point of the interspecific discriminant vector. The ANOVA data are placed in the line between the compared data sets.

	Distance from zero
Potential contact zone (total 84, *crassispinus* 34, *crasecundus* 50)	1.6173 ± 1.0000 [0.1186.3.8912]
ANOVA [F, p]	0.370, 0.543
Allopatric zones (total 119, *crassispinus* 85, *crasecundus* 34)	1.6941 ± 0.7906 [0.0326,3.4717]
ANOVA [F, p]	11.267, 0.001
Syntopic sites (total 31, *crassispinus* 18, *crasecundus* 13)	1.1465 ± 0.8740 [0.1186,3.4894]
ANOVA [F, p]	12.383, 0.001
Potential contact zone without syntopic sites (total 53, *crassispinus* 16, *crasecundus* 37)	1.8927 ± 0.9727 [0.1258,3.8912]

We conclude that *Temnothorax crassispinus* and *Temnothorax crasecundus* sp. n. are a pair of cryptic, parapatric species showing hybridization in the contact zone. We follow here the Pragmatic Species Concept of Seifert (2014) which allows local hybridization and even weak introgression between species as long as they are separable over their whole range in the vast majority of samples. Seifert formulated his concept in several sentences: “*A species is a cluster of organisms which passed a threshold of evolutionary divergence. Divergence is determined by one or several operational criteria described by an adequate numerics. A single conclusive operational criterion is sufficient. Conflicts between operational criteria require an evolutionary explanation. Thresholds for each operational criterion are fixed by con sensus among the experts of a discipline under the principle of avoiding over-splitting. Clusters must not be the expression of intraspecific polymorphism.*”

Seifert proposed as operational criterion for the discipline “multivariate investigation of ant worker morphology” and as a remedy against over-splitting that at least 97% of the classifications by exploratory data analyses should agree with the classifications by linear discriminant analyses that form the final species hypothesis. However, 4% disagreement or 96% confirmation seem to be a more reasonable threshold when considering the performance of the two most powerful methods of NC-clustering, NC-K-Means and NC-Ward, in the separation of 74 cryptic ant species (Table 1 in Seifert et al. 2013).

The consequences of threshold decisions may be illustrated by an example. Over many years we studied the situation in the Mediterranean *Temnothorax lichtensteini* complex and intended to accept three species: the largely western entity *Temnothorax lichtensteini* (Bondroit, 1918), a second, largely eastern, semipatric entity (provisionally designated by Seifert (2007) in his key as “*Temnothorax lichtensteini* sp. 2”) and a third entity restricted to southern Balkans. Csösz et al. (2013) showed that a linear discriminant analysis could separate all three clusters. Yet, the agreement between NC-clustering and the LDA was only 92% in the second entity but 100% in the third entity. As a consequence, a taxonomic naming of the second entity was suspended whereas the third one was described as the new species *Temnothorax laconicus* Csösz et al. 2013. It is possible that multisource taxonomy, in particular application of adequate nuDNA markers, could lead to other conclusions in case of the second entity.

We want to emphasize in this context that hybridization and reticulate evolution is a matter of fact in the living nature around us (reviewed, e.g., in Abbott et al. 2013). Exemplary groups in animals are ducks (Phillips 1915, 1921), redstarts (Ertan 2002) or butterflies (Mavárez et al. 2006, Kronforst 2008). Ants are no exception: as much as 18% of the Central European ant species are credibly shown to hybridize (Pearson 1983, Seifert 1984, Douwes and Stille 1991, Seifert 1999, 2006, Seifert et al. 2010, Kulmuni et al. 2010, Steiner et al. 2011, Van der Have et al. 2011, Bagherian et al. 2012, Seifert 2013). A workable species concept has to recognize this. Alternatively, a taxonomist considering the term “reproductive isolation” in its genuine meaning of an impenetrable barrier would have to synonymize a big portion of taxa currently considered by any taxonomist as well-separable species.

Finally we address the question if our two entities might represent an intraspecific dimorphism instead of representing different species by considering geographic distribution and the frequency and distribution of supposedly mixed nests in the sympatric and allopatric zones. Frequencies of discrete morphs provided by the gene pool of a single species may show steep geographic gradients - remind alone of the famous text book example of the Peppered Moth *Biston betularia* (e.g., Cook 2003). However, the clear-cut parapatric partitioning as in our case is not known for morphs. This is the primary, zoogeographic argument against intraspecific polymorphism. Considering the 84 nest samples from the sympatric area from Greece north to Moldova and accepting only classifications with posterior probabilities of p>0.90, we found only two nests (=2.4%) putatively containing both *Temnothorax crassispinus* and *Temnothorax crasecundus* workers morphs whereas 97.6% contained pure samples of either species. According to these two arguments, we consider intraspecific dimorphism with panmictic behavior as extremely unlikely. Even more, these two putatively mixed nests in the sympatric zone could represent hybrids or misidentifications rather than indicating mixtures of pure phenotypes. A small percentage of misidentification is always expectable in a large material of 203 samples with 603 workers and becomes obvious in a third “mixed” sample from near Hohburg / Saxony. This site is deeply within the *Temnothorax crassispinus* range and some 1350 km away from the next site of *Temnothorax crasecundus* sp. n. – occurrence of a mixed nest should be impossible there.

### Species description

#### Temnothorax crasecundus sp. n.

Taxon classificationAnimaliaHymenopteraFormicidae

http://zoobank.org/9C3131DE-FCCE-4078-A0BB-2BFC68EBA7B5

##### Etymology.

In the provisional internal naming system of the senior author the new species had been designated over the years as “*Temnothorax crassispinus* sp. 2”. The taxonomic name, composed of “cra” (first syllable of crassispinus) and “secundus” (= the second), intends to indicate both this history and the close relationship.

##### Type material.

See above under “Material”.

##### Description of the worker caste.

All morphometric data given in the following verbal description are arithmetic means of 256 worker individuals calculated by fusing Seifert’s and Csösz’s data sets. Harmonization of the different data sets has been performed by the function CW = 1.0791 * CWb.

**Figure 12.**
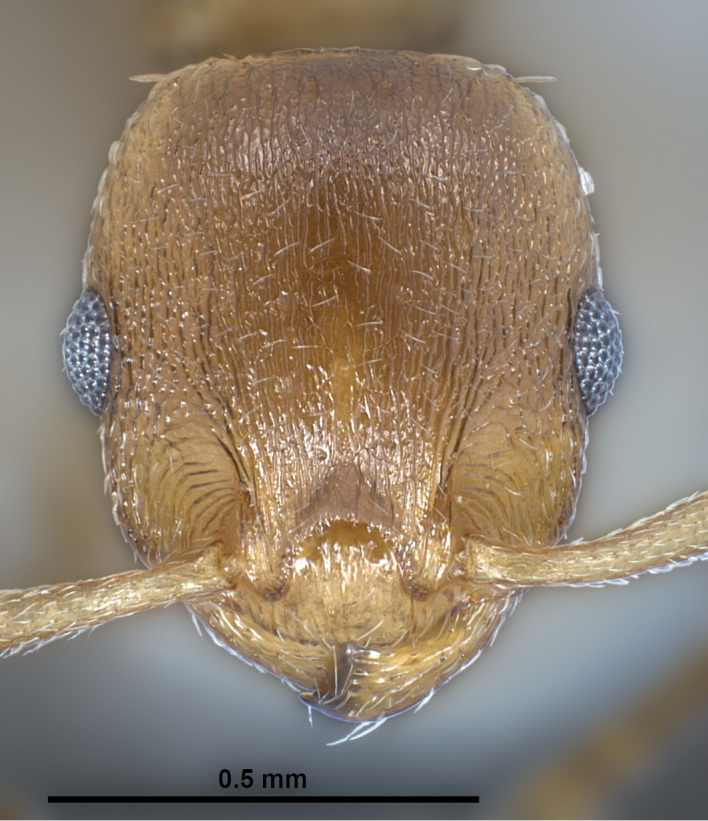
*Temnothorax crasecundus* sp. n. Head of holotype.

**Figure 13.**
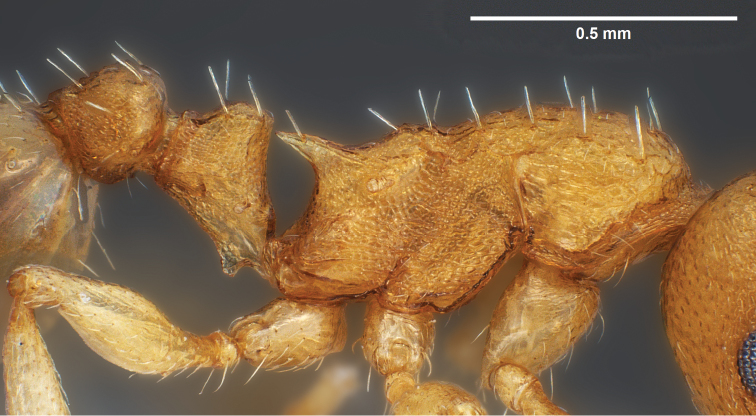
*Temnothorax crasecundus* sp. n. Lateral aspect of holotype.

**Figure 14.**
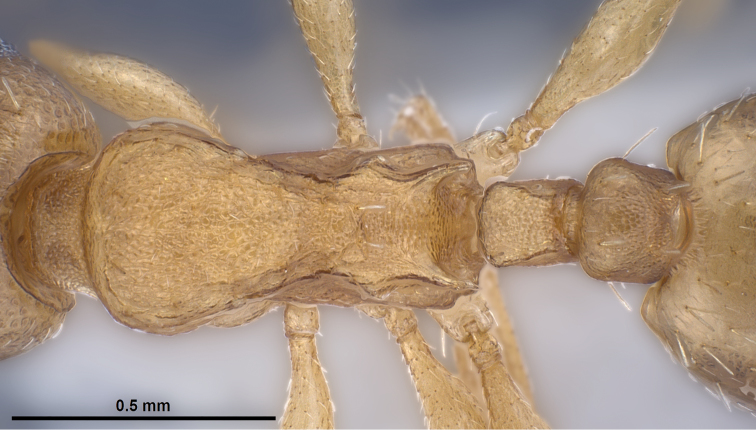
*Temnothorax crasecundus* sp. n. Dorsal aspect of holotype.

**Figure 15.**
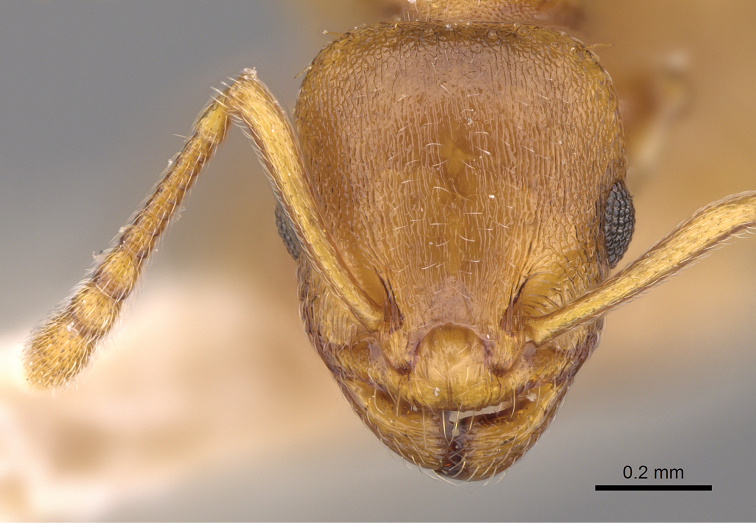
*Temnothorax crassispinus* (Karavajev). Head of a syntype.

**Figure 16.**
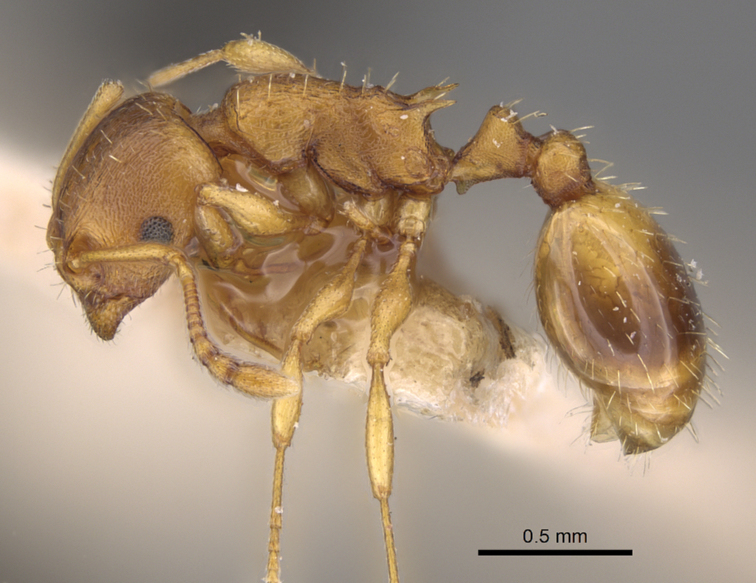
*Temnothorax crassispinus* (Karavajev). Lateral aspect of a syntype.

**Figure 17.**
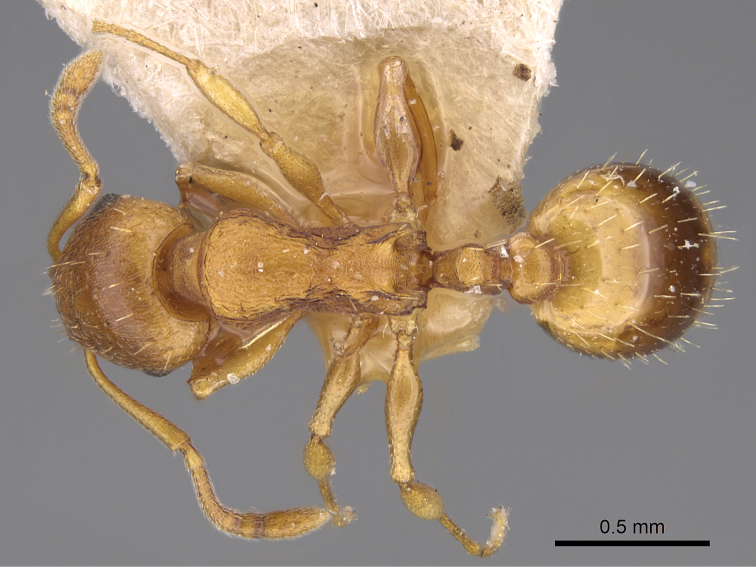
*Temnothorax crassispinus* (Karavajev). Dorsal aspect of a syntype.

*Worker* (Tables 2, 3; Figs 12–14; compare with photos of *Temnothorax crassispinus* type in Figs 15–17): Medium-sized species (CS 641 µm, ML 760 µm). Head weakly elongated but significantly more than in *Temnothorax crassispinus* (CL/CW 1.069 vs. 1.055, CL/CWb 1.153 vs. 1.139). Head in dorsal aspect with strongly convex postocular sides, convex genae and straight posterior margin. Eyes with a moderate distance from posterior margin of vertex (PoOc/CL 0.391) and medium-sized in terms of the genus but significantly smaller than in *Temnothorax crassipinus* (EYE/CS 0.210 vs. 0.216). Scape moderately long (SL/CS 0.762, SL/CSb 0.793) and with variable pubescence: with the scape directed caudad, pubescence is appressed to decumbent (0–15°) at inner margin and more subdecumbent (30°) at outer margin. Frontal carinae rather distant (FRS/CS 0.361), their median part more or less parallel. Sculpture on central vertex regularly longitudinally carinulate. A transversal line between the frontal carinae, positioned immediately posterior of the frontal triangle, crosses 21–23 carinulae. A small longitudinal zone on median vertex occasionally without sculpture and shining. Lateral vertex and head with a more irregular sculpture, being a mixture of microreticulate and rugulose structures. Antennal sockets on shining ground surrounded by 7–8 concentric rugulae. Clypeus between sagittal level of frontal carinae rather smooth but with 5–8 longitudinal carinulae. Frontal triangle with very delicate microsculpture and 0–4 longitudinal carinulae.

**Table 2.** Data set of Seifert; nest sample means of morphometric data of the workers of the cryptic species *Temnothorax crasecundus* sp. n. and *Temnothorax crassispinus* (Karavajev, 1926). Arrangement of data: arithmetic mean ± standard deviation [minimum, maximum]. F values and significance levels p are from an univariate ANOVA; the F values of the most separating characters are given in heavy type.

	*Temnothorax crasecundus* sp. n. (n=29)	ANOVA F, p	*Temnothorax crassispinus* (n=75)
CS	641 ± 36 [594,713]	0.02, n.s.	645 ± 30 [556,713]
CL/CW	1.065 ± 0.014 [1.038,1.087]	16.90, 0.000	1.053 ± 0.013 [1.013,1.081]
SL/CS	0.767 ± 0.011 [0.741,0.786]	1.38, n.s.	0.764 ± 0.013 [0.734,0.790]
PoOc/CL	0.395 ± 0.007 [0.380,0.406]	3.63, n.s.	0.392 ± 0.007 [0.373,0.405]
EYE/CS	0.210 ± 0.005 [0.201,0.219]	29.22, 0.000	0.216 ± 0.005 [0.207,0.233]
FRS/CS	0.361 ± 0.008 [0.344,0.376]	2.48, n.s.	0.364 ± 0.008 [0.345,0.386]
SPBA/CS	0.280 ± 0.010 [0.252,0.297]	26.53, 0.000	0.294 ± 0.012 [0.268,0.322]
SPTI/CS	0.325 ± 0.014 [0.295,0.347]	**75.95**, 0.000	0.350 ± 0.013 [0.324,0.377]
SPST/CS	0.285 ± 0.014 [0.250,0.308]	**179.20**, 0.000	0.323 ± 0.014 [0.286,0.354]
SP/CS	0.218 ± 0.015 [0.188,0.246]	**202.63**, 0.000	0.260 ± 0.012 [0.232,0.290]
PEW/CS	0.253 ± 0.007 [0.242,0.270]	6.76, 0.011	0.258 ± 0.008 [0.239,0.275]
PPW/CS	0.353 ± 0.010 [0.335,0.375]	24.77, 0.000	0.367 ± 0.013 [0.331,0.395]
PEH/CS	0.357 ± 0.008 [0.343,0.374]	14.22, 0.000	0.364 ± 0.009 [0.343,0.391]
PEL/CS	0.472 ± 0.011 [0.452,0.497]	7.41, 0.008	0.479 ± 0.013 [0.446,0.508]
ML/CS	1.187 ± 0.016 [1.158,1.221]	0.08, n.s.	1.188 ± 0.015 [1.156,1.239]
MW/CS	0.605 ± 0.010 [0.580,0.628]	3.68, n.s.	0.600 ± 0.011 [0.780,0.626]
MH/CS	0.524 ± 0.011 [0.500,0.547]	11.93, 0.001	0.532 ± 0.012 [0.507,0.566]
MPGR/CS	2.10 ± 0.56 [1.31,3.41]	0.25, n.s.	2.04 ± 0.49 [1.07,3.23]

**Table 3.** Data set of Csösz; nest sample means of morphometric data of the workers of the cryptic species *Temnothorax crasecundus* sp. n. and *Temnothorax crassispinus* (Karavajev, 1926). Arrangement of data: arithmetic mean ± standard deviation [minimum, maximum]. F values and significance levels p are from an univariate ANOVA; the F values of the most separating characters are given in heavy type.

	*Temnothorax crasecundus* sp. n. (n=55)	ANOVA F, p	*Temnothorax crassispinus* (n=44)
CSb	614 ± 37 [539,718]	1.73, n.s.	623 ± 36 [544,688]
CL/CWb	1.155 ± 0.018 [1.120,1.196]	14.03, 0.000	1.140 ± 0.021 [1.082,1.180]
SL/CSb	0.791 ± 0.013 [0.763,0.836]	6.40, 0.013	0.784 ± 0.013 [0.756,0.811]
PoOc/CL	0.390 ± 0.007 [0.379,0.405]	0.00, n.s.	0.390 ± 0.007 [0.370,0.410]
EL/CSb	0.255 ± 0.007 [0.236,0.279]	0.71, n.s.	0.256 ± 0.005 [0.247,0.268]
FRS/CSb	0.375 ± 0.008 [0.358,0.397]	1.57, n.s.	0.377 ± 0.008 [0.362,0.400]
MW/CSb	0.630 ± 0.010 [0.610,0.662]	1.37, n.s.	0.627 ± 0.017 [0.590,0.678]
ML/CSb	1.227 ± 0.017 [1.193,1.268]	1.01, n.s.	1.223 ± 0.023 [1.183,1.303]
SPBA/CSb	0.298 ± 0.012 [0.277,0.321]	32.89, 0.000	0.312 ± 0.013 [0.282,0.339]
SPTI/CSb	0.333 ± 0.016 [0.300,0.384]	**96.86**, 0.000	0.365 ± 0.016 [0.332,0.397]
SPWI/CSb	0.352 ± 0.018 [0.295,0.405]	**110.18**, 0.000	0.388 ± 0.016 [0.355,0.422]
SPST/CSb	0.289 ± 0.015 [0.253,0.319]	**220.08**, 0.000	0.330 ± 0.011 [0.298,0.356]
SPL/CSb	0.162 ± 0.006 [0.149,0.175]	17.06, 0.000	0.157 ± 0.008 [0.137,0.175]
PEW/CSb	0.269 ± 0.008 [0.252,0.288]	10.18, 0.002	0.275 ± 0.011 [0.255,0.317]
PEH/CSb	0.372 ± 0.009 [0.354,0.398]	7.88, 0.006	0.377 ± 0.009 [0.361,0.400]
NOH/CSb	0.169 ± 0.007 [0.156,0.202]	29.11, 0.000	0.177 ± 0.007 [0.164,0.189]
NOL/CSb	0.254 ± 0.010 [0.232,0.274]	4.68, 0.033	0.258 ± 0.008 [0.237,0.275]
NODL/CSb	0.296 ± 0.014 [0.266,0.318]	0.02, n.s.	0.295 ± 0.017 [0.267,0.340]
PPW/CSb	0.368 ± 0.009 [0.348,0.387]	19.17, 0.000	0.377 ± 0.012 [0.348,0.405]
PPH/CSb	0.350 ± 0.008 [0.335,0.368]	7.74, 0.007	0.356 ± 0.011 [0.340,0.384]
PPL/CSb	0.255 ± 0.009 [0.234,0.273]	1.04, n.s.	0.257 ± 0.008 [0.236,0.272]
PL/CSb [%]	0.413 ± 0.011 [0.383,0.434]	5.74, 0.019	0.419 ± 0.013 [0.393,0.454]

Mesosoma moderately wide (MW/CS 0.608) and metanotal depression always developed (MpGr/CS 2.1%). Propodeal spines rather long and acute but distinctly shorter than in *Temnothorax crassipinus* (SPST/CS 0.283 vs. 0.322). Distance of their bases and tips rather large but significantly smaller than in *Temnothorax crassipinus* (SPBA/CS 0.286 vs. 0.300, SPTI/CS 0.324 vs. 0.353), spine tips slightly curving inwards. Direction of spines in lateral view deviating from longitudinal axis of mesosoma by 26–29°. Mesosoma irregularly microreticulate-rugulose with few superimposed longitudinal rugae on promesonotum. Metapleuron more regularly longitudinally carinate-rugose.

Petiole in lateral view rather high and with a weakly concave frontal face; the anterior profiles of node and peduncle form an angle of about 150–155° whereas anterior and dorsal profiles of node form an angle of 90–105°. Dorsal profile of node weakly convex or nearly straight and moderately long, steeply sloping down to caudal cylinder. The profiles of this slope and of the caudal cylinder form an angle of about 140°. Whole surface of petiolar and postpetiolar nodes microreticulate with a mesh width of 9–13 µm. Two longitudinal rugae typically demarcate the margin of dorsal petiolar plane while the sides of petiolar tergites are stabilized by one longitudinal carina/ruga on each side.

Overall body color dirty yellow to light brown with a strong yellowish component. Mesosoma, appendages, waist and basis of first gaster tergite usually lighter yellow to dirty yellow. Head dorsum and the posterior surfaces of gaster tergites usually darker, generally yellowish brown. Lighter heads occur.

##### A more simple means for species delimitation.

There is considerable overlap in each of the 29 shape characters and absolute size (Tables 2 and 3) and a much larger one on individual level (data not shown). This excludes a simple separation of *Temnothorax crasecundus* sp. n. and *Temnothorax crassispinus* by single characters. The complex species delimitation procedures presented above require much training of the investigator and a high-quality optical equipment. Even then, data recording for a single nest sample composed of three workers needs 100–120 minutes. In order to allow a practitioner of biodiversity or ecosystem research a more easy approach to the problem, we developed a more simplified determination rule using six absolute measurements. With all measurements given in mm, the discriminant

D(6) = 22.058*PoOc+17.640*SL-66.166*SPST+38.233*MW-28.926*PPW -35.873*SPTI-1.797

classified 203 nest samples with an error of 3.4%. Samples with an arithmetic mean of D(6) < 0 are determined as *Temnothorax crassispinus* and those with larger values as *Temnothorax crasecundus* sp. n. With PoOc, SL and SPST being recorded bilaterally, a trained investigator needs for the resulting nine measurements about 15 minutes per individual.

The most simple means for separation of *Temnothorax crasecundus* from *Temnothorax nylanderi* is geography: the shortest distance between a site of both species is 1000 km and a closing of this broad gap is prevented by habitat saturation of the omnipresent, highly competitive *Temnothorax crassipinus*. A rather simple phenotypical species delimitation is possible using three absolute measurements. With all measurements given in mm, the discriminant

D(3) = 129.53*SPBA–120.88*PPW+133.8*MpGR +4.446

classified 87 nest samples with an error of 3.4%. Samples with an arithmetic mean of D(3) < 0.64 are determined as *Temnothorax nylanderi* and those with larger values as *Temnothorax crasecundus* sp. n.

##### Zoogeography and biology.

The present zoogeography (Fig. 11) suggests that *Temnothorax crasecundus* sp. n. survived the last glaciation in an Aegean / West Anatolian arboreal refuge centre near to sea level. The refuge of *Temnothorax crassispinus* should have been situated rather close to that area in the lowlands of the West Balkans along the eastern Adria and was divided from the *Temnothorax crasecundus* sp. n. refuge by the Dinaric and Greek mountains. Unhindered by its competing sibling species, there was probably a fast postglacial spreading of *Temnothorax crasecundus* sp. n. to the north and northeast over Bulgaria, Romania, Moldova and the southern Ukraine to Caucasia and to the east over Asia Minor to Transcaucasia. Spreading to the west was blocked by a front-line confrontation with *Temnothorax crassispinus* – the underlying mechanisms stabilizing this parapatry are probably comparable to those along the Central European front line between *Temnothorax nylanderi* and *Temnothorax crassispinus* (Pusch et al. 2006). Postglacial spreading of *Temnothorax crassispinus* was rapid in northern and northeastern direction. The southern limit of its distribution in the Ukraine and south Russia coincides with the southern border of the natural range of the woodland steppe (Bohn et al. 2000). Despite a larger distance of its Pleistocene refuge from wintercold continental areas of European Russia, *Temnothorax crassispinus* obviously arrived here before *Temnothorax crasecundus*. This colonization advantage is probably explained by a higher freezing resistance: the most wintercold known site in *Temnothorax crassipinus* near Kazan / Russia has a mean January temperature of –13 °C and that of *Temnothorax crasecundus* near Erzurum / East Anatolia one of –10.5 °C (climatic data from www.weather-and-climate.com). Biology and ecology of *Temnothorax crasecundus* sp. n. are not studied in detail. Nests were found on ground of deciduous or coniferous forests in microspaces such as hollow acorns, nuts, rotten twigs or galls. Nest populations are monogynous.

## Supplementary Material

XML Treatment for Temnothorax crasecundus
